# Randomized study comparing full dose monotherapy (S-1 followed by irinotecan) and reduced dose combination therapy (S-1/oxaliplatin followed by S-1/irinotecan) as initial therapy for older patients with metastatic colorectal cancer: NORDIC 9

**DOI:** 10.1186/s12885-017-3526-8

**Published:** 2017-08-16

**Authors:** Stine Braendegaard Winther, Pia Österlund, Åke Berglund, Bengt Glimelius, Camilla Qvortrup, Halfdan Sorbye, Per Pfeiffer, - on behalf of the Academy of Geriatric Cancer Research (AgeCare)

**Affiliations:** 1 Department of Oncology, Odense University Hospital, Sdr. Boulevard 29, 5000 Odense C, Denmark; 2 Department of Oncology, Helsinki University Central Hospital, Stenbäckinkatu 9, PO BOX 100, FI-00029 Helsinki, Finland; 3 Clinicum, Helsinki University, Haartmaninkatu 8, 3th floor, PO BOX 63, 00014 Helsinki, Finland; 4 Department of Immunology, Genetics and Pathology, Uppsala University, Dag Hammarskjölds väg 20, 751 85 Uppsala, Sweden; 5 Department of Oncology and Department of Clinical Science, Haukeland University Hospital, Postboks 1400, 5021 Bergen, Norway

**Keywords:** Randomized phase II, Non-intensive, SOX, IRIS, Oral, Geriatric assessment, Metastatic colorectal cancer

## Abstract

**Background:**

Metastatic colorectal cancer (mCRC) is a disease of older age, but there is a relative lack of knowledge about effects of chemotherapy in older patients as they are under-represented in clinical trials. Little data can guide whether the strategy in older mCRC patients should be a sequential full-dose monotherapy chemotherapy approach or a dose-reduced combination chemotherapy approach. The oral 5FU prodrug S-1 seems to have less side effects than capecitabine and should be an optimal drug for older patients, but few data are available. Improved geriatric assessments are needed to select which older patients should receive therapy.

**Methods:**

The NORDIC 9 trial is a Nordic multicenter randomized phase II study comparing full dose monotherapy (S-1 30 mg/m^2^ twice daily days 1–14 every 3 weeks, followed by second line irinotecan 250–350 mg/m^2^ iv day 1 every 3 weeks or 180–250 mg/m^2^ iv day 1 every 2 weeks) with reduced dose combination therapy (S-1 20 mg/m^2^ days 1–14 + oxaliplatin 100 mg/m^2^ iv day 1 every 3 weeks, followed by second line S-1 20 mg/m^2^ days 1–14 + irinotecan 180 mg/m^2^ day 1 every 3 week) for older patients (≥70 years) with mCRC who are not candidates for full-dose standard combination therapy. Additional bevacizumab (7.5 mg/kg) is optional in first-line. Blood samples and tumor tissue will be collected to investigate predictive markers. Geriatric screening tools (G-8, VES-13, Timed-Up-and-Go and Handgrip strength), Charlson Comorbidty Index and quality of life (EORTC QLQ-C30) will be evaluated as predictors of efficacy and toxicity. The target sample size is 150 patients.

The primary endpoint is progression-free survival and secondary endpoints are time-to-failure of strategy, overall survival, response rate, toxicity, and correlations between biomarkers, pre-treatment characteristics and geriatric assessments.

**Discussion:**

The study will add knowledge on how to treat older mCRC patients who are not candidates for standard combination therapy. Furthermore it may provide understanding of efficacy and tolerability of chemotherapy in older cancer patients and thus offer a better chance for tailored treatment strategies in these patients.

**Trial registration:**

EU Clinical Trial Register, EudraCT no. 2014–000394-39. Registered 05 May 2014.

## Background

Survival of patients with metastatic colorectal cancer (mCRC) has increased considerably over the past several decades. Contributory factors for this improvement are surgical resection or other ablative techniques for metastatic disease, a more strategic approach to the delivery of systemic therapy with a continuum of care, better selection of patients to different treatments and more lines of effective anticancer drugs [1].

The backbone of medical treatment of mCRC is 5-fluorouracil (5-FU). Since about two decades, it is seldom given alone but biochemically modulated with calcium folinate due to higher response rates and likely improved survival [2]. The oral prodrugs of 5-FU (capecitabine, UFToral and S-1) are as efficient as modulated 5-FU and efficacy is further increased when 5-FU is combined with irinotecan or oxaliplatin [1, 3]. Combination regimens are often the best first-line choice, but monotherapy is an alternative in some clinical situations based upon the results of several trials. In both the CAIRO [4], FOCUS1 [5] and FFCD 2000–05 trials [6] unselected patients with previously untreated mCRC were randomized between a sequential strategy and combination therapy. In the FOCUS2 [7] and FFCD 2001–02 [8] trials, the doublet strategy in older patients with mCRC was explored, in FOCUS2 with a population of patients who were not candidates for full-dose chemotherapy. All studies showed that it is safe to start monotherapy if followed by new therapy upon progression.

Median overall survival (OS) often exceeds 24 months for mCRC patients included in clinical trials. In unselected populations of mCRC patients, however, median OS is only around 12 months [9–11] although, in one of the population-based studies [12], median OS was 21.3 months in the subgroup of patients (36%) treated with combination chemotherapy as in a clinical trial. The short OS in general populations of mCRC patients is mainly for a short survival in patients above 70–75 years of age and in patients not receiving any chemotherapy due to a variety of reasons [12, 13]. The reported OS improvement over time in mCRC patients is mainly seen in younger patients and only minor improvements are found in older patients [14]. In a large community-based study, older patients (65+ years) were less likely to receive first-line doublet chemotherapy and also less likely to receive irinotecan, oxaliplatin, and bevacizumab during the entire course of the disease [15].

In a combined analysis of more than 2500 patients treated with different irinotecan/5-FU schedules in four first-line phase III trials, the authors concluded that older patients (70+ years) who fulfilled the inclusion criteria of the trials had similar benefits of treatment and similar risk of toxicity as younger patients, and these results have been confirmed in other studies including systematic reviews [16–19]. A comparable outcome in patients below or above 70 years included in randomized trials is reasonably explained by inclusion of only the very fittest older patients, based on strict inclusion criteria, e.g. appropriate performance status and sufficient organ functions, but these patients are not representative of the majority of older patients cared for in the oncological clinics [20]*.* This has an impact on our perception of standard therapy, as colorectal cancer (CRC) is a disease of older age with approximately 50% of the patients being 70 years or older [9, 21]. From 2001 to 2005 the median age of patients with mCRC included in clinical trials was only 62 years [22], while the median age at mCRC diagnosis is 71–74 years according to cancer registries [14].

In clinical practice many oncologists recommend full-dose monotherapy or reduced dose combination therapy (without much evidence) in older and/or frail patients. In the FOCUS1 trial [5], where single agent therapy versus combination chemotherapy was explored, the median age was only 64 years, despite permissive entry criteria and no upper age limit, and a retrospective survey showed that almost twice as many patients had been treated off-trial during the same period, frequently using reduced-dose or single-agent schedules. The most frequent reasons for non-inclusion were physicians’ concerns about the adverse effects of standard-dose treatments and patients’ wishes to avoid toxic effects. The authors therefore designed FOCUS2 [7] for patients where the treating oncologist considered standard full-dose regimens to be unsuitable. A total of 459 patients (median age 74 years) were randomized to reduced dose monotherapy or reduced dose oxaliplatin combination therapy, with a planned dose escalation in patients with no or few side effects. However, dose escalation was only effectuated in one third of the patients. Combination therapy more than doubled response rate (from 13% to 35%) and marginally prolonged progression-free survival (PFS) (HR 0.84 (0.69–1.01), *p* = 0.07), but OS was not improved (HR 0.99 (0.81–1.18)).

In a recent French phase III trial (FFCD-2001-02), where 282 mCRC patients aged ≥75 years received either first-line monotherapy with 5-FU or FOLFIRI, no significant differences in PFS (5.2 months vs. FOLFIRI 7.3 months, HR 0.84 (0.66–1.07), *p* = 0.15) or OS (14.2 months vs. 13.3 months, HR 0.96 (0.75–1.24)) were found [8]. In line with these data, a recent meta-analysis showed that combination chemotherapy prolonged PFS but not OS with an increased risk of toxicity in older patients [23].

Further development of antineoplastic drugs may lead to agents with a potentially more favorable toxicity profile which may be of benefit especially for older cancer patients. S-1 (Teysuno®) is a third generation oral fluoropyrimidine comprised of tegafur, a pro-drug of 5-FU, and two modulators of 5-FU metabolism; gimeracil and oteracil [24]. S-1 provides sustained 5-FU plasma concentrations with reduced toxicities like hand-foot-syndrome (HFS) and probably cardiotoxicity and diarrhoea, due to the addition of the modulators [25–27]. Randomized Asian studies in mCRC patients have shown that S-1 is as effective as 5-FU or capecitabine as monotherapy, and in combination with oxaliplatin [28, 29] or irinotecan [30]. In Japan S-1 is approved for the treatment of several cancers including gastric, colorectal and pancreatic cancers, but in the European Union presently only for gastric cancer [24].

Because of higher activity of CYP2A6 and thereby more effective conversion of S-1 to 5-FU in Caucasians, the optimal dose is lowered in Caucasians compared to Asians [24, 26]. In an observational chart review the use of S-1 as monotherapy (30 mg/m^2^ twice daily on days 1–14 every 3 weeks) and in combination (25 mg/m^2^ twice daily on days 1–14 every 3 weeks) with oxaliplatin or irinotecan, respectively, in 71 older mCRC patients was evaluated [26]. In all three settings S-1 was well-tolerated with a low rate of clinical and haematological adverse events, and especially a very low incidence of HFS was found. Furthermore the treatment indicated comparable efficacy to standard regimens in mCRC. However further prospective Western studies are needed to confirm efficacy and safety of S-1 in young and older populations.

Bevacizumab has in randomized studies, as a part of combination therapy improved efficacy in mCRC patients [31–34]. Retrospective cohort studies indicate a similar benefit on a population level [35, 36]. In a recent study, S-1 plus bevacizumab therapy was effective and safe for older (median age 75 years) mCRC patients [37]. In the SALTO trial, S1 plus bevacizumab was as effective as capecitabine plus bevacizumab with a significantly lower incidence of HFS [38].

Based on these facts, the ongoing NORDIC 9 trial was initiated to compare full dose single agent S-1 therapy and dose-reduced combination therapy in older patients not considered candidates for standard combination therapy, hypothesizing that reduced combination therapy improves efficacy compared to fulldose monotherapy.

### Geriatric assessments

A major challenge in the treatment of older cancer patients today is to select the patients who can tolerate and benefit from treatment. Comorbidity, polypharmacy, different state of nutrition, functional and psychosocial capabilities including cognition are all elements of pivotal character when an overall assessment of the older patient is made [39]. Several attempts to develop tools for this evaluation have been made. The Comprehensive Geriatric Assessments (CGA) is recommended by the International Society of Geriatric Oncology (SIOG) [40, 41], but CGA is too time-consuming to be performed in a routine oncological clinical setting. Therefore several screening tools to identify the patients, who would benefit from a CGA and potential treatment, have been tested. In the NORDIC 9 trial, we have chosen some of the most promising and least time-consuming tests (G-8, VES-13, Timed-Up-and-Go and Handgrip strength) to evaluate their predictive and prognostic value.

- The G-8 is an 8-item screening tool including nutrition, mobility, neuropsychological problems, number of medications, self-perception of health and age [42]. The G-8 is scored by a nurse and takes approximately 5 min.
- The Vulnerable Elders Survey (VES-13) is a 13-item self-administered instrument based upon age, self-rated health and the ability to perform physical and functional activities. It predicts functional decline and mortality [43, 44] and takes approximately 5 min to fill out.
- Timed-up-and-go-test (TUG) assesses the physical status of the older patient. The patient is observed and timed while he/she rises from an armchair, walks 3 metres, turns, walks back, and sits down again [45].
- Handgrip strength (GS) is measured with a hand dynamometer and reflects the upper extremity strength. It correlates to patients’ overall muscle strength, bone density, nutrition status and frailty [46].

Furthermore

- Charlson Comorbidity Index is scored by a physician and takes approximately 5 min.
- Quality of Life (QoL) by EORTC-QLQ-C30 is completed at baseline and after 3rd and 6th course of treatment, to supplement the overall assessment.

## Methods/design

### Design

**Fig.1:**
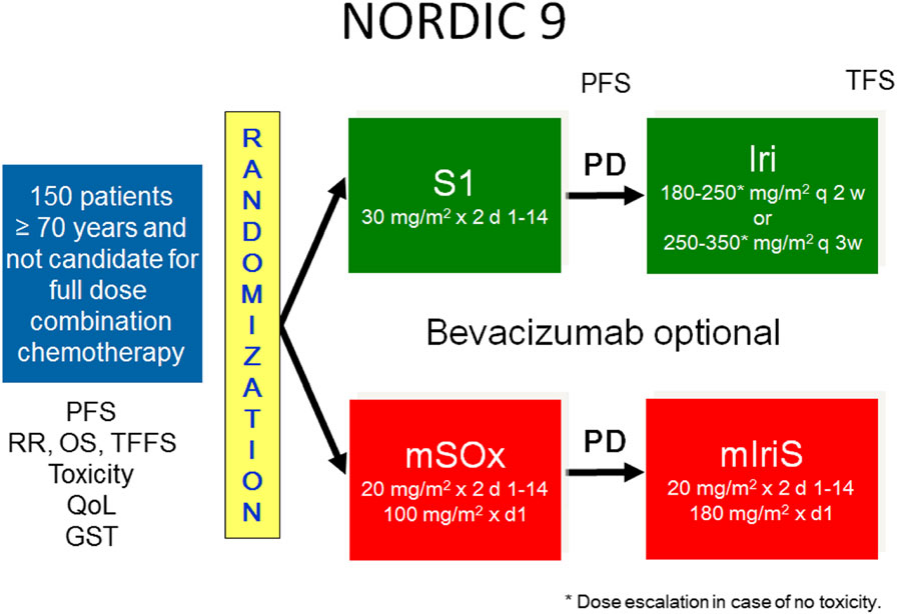
Study design of the NORDIC 9 trial

NORDIC 9 is an open multicenter randomized phase II trial aiming at investigating the efficacy of full dose monotherapy (S-1 followed by second line irinotecan upon progression) and a reduced dose combination therapy (S-1/oxaliplatin followed by second line S-1/irinotecan) as initial therapy for older (≥70 years) mCRC patients who are not candidates for full dose standard combination chemotherapy as evaluated by the treating physician (Fig. 1). Additional therapy with bevacizumab in first-line setting is optional, but that decision should be made before randomization.

### Participants

One hundred and fifty patients will be enrolled at 20 oncologic departments in Finland, Norway, Sweden and Denmark. All patients must be 70 years or older, have histologically verified colorectal adenocarcinoma, non-resectable mCRC and not be candidate for standard full-dose combination chemotherapy as evaluated by the treating physician. Other inclusion criteria are WHO performance status 0–2, life expectancy of at least 3 months, no prior chemotherapy except adjuvant fluoropyrimidine therapy completed more than 180 days before randomization, no evidence of CNS metastasis, and adequate hematological, kidney (GFR > 30 ml/min, but if calculated GFR is ≤70 ml/min, GFR must be measured) and liver function.

The enrollment started in March 2015 and target recruitment period is estimated to be 24 months and follow-up period/end date is estimated to be 6 months after inclusion of the last patient. Randomization will be conducted as a block randomization stratified according to institution and planned therapy with bevacizumab. All patients must provide written informed consent.

### Treatment

Patients will be randomized to receive:

- Arm A: Full dose single agent strategy
  - *First-line:* S-1 30 mg/m^2^ twice daily days 1–14 every 3 weeks followed upon progressive disease (PD) by
  - *Second line:* Irinotecan 250 mg/m^2^ iv day 1 every 3 weeks or irinotecan 180 mg/m^2^ iv day 1 every 2 weeks (optional). In the absence of toxicity (except alopecia) above grade 1, it is recommended to increase the irinotecan dose in steps to 350 mg/m^2^ iv day 1 every third week or irinotecan 250 mg/m^2^ iv day 1 every second week.
- or
- Arm B: Reduced dose (80%) combination-therapy strategy
  - *First-line:* S-1 20 mg/m^2^ twice daily days 1–14 and oxaliplatin 100 mg/m^2^ iv day 1 every 3 weeks followed upon PD by
  - *Second line:* S-1 20 mg/m^2^ twice daily days 1–14 and irinotecan 180 mg/m^2^ iv day 1 every 3 weeks

Bevacizumab (7.5 mg/kg iv day 1) may be added to first-line chemotherapy (monotherapy or combination) at the discretion of the treating physician.

Blood samples (serum and EDTA plasma) and tumor tissue will be collected for future investigations of biomarkers.

The Geriatric screening tools (GST) (G-8, VES-13, Timed-Up-and-Go, Handgrip strength), Charlson Comorbidity Index and QoL will be completed before randomization.

#### Dose modifications

In patients with GFR 30–49 ml/min, the dose of S-1 must be reduced with 5 mg/m^2^ (from 30 mg/m^2^ to 25 mg/m^2^ or from 20 mg/m^2^ to 15 mg/m^2^).

When dose reduction is needed during therapy because of toxicity, the dose of S-1 will be reduced with 5 mg/m^2^ (from 30 mg/m^2^ to 25 mg/m^2^ or from 20 mg/m^2^ to 15 mg/m^2^) and the dose of oxaliplatin or irinotecan will be reduced with 25%.

#### Duration of therapy

Treatment is recommended until progression, unacceptable side-effects, patients’ wish of ending treatment or patients’ wish for chemo-holiday. After disease progression, the patients will be offered second-line therapy, which will continue until new progression.

#### Evaluation of treatment delivery

After every 9 weeks a CT-scan will be performed to evaluate time of progression in all patients and radiological response in patients with measurable disease (according to RESIST criteria 1.1).

Post treatment evaluation will be assessed by clinical and radiological tumor evaluation every 2 months until second progression is diagnosed.

#### Safety

Adverse events will be evaluated according to NCI-CTC version 4.0 for all patients for 28 days following the last dose of study drug.

When 50 patients are included, a safety analysis (toxicity and dose-intensity) after the three first cycles of therapy will be conducted and evaluated by the protocol committee to ensure tolerability. Inclusion of patients may continue during safety analysis.

### Study objectives

The primary endpoint is PFS. Secondary objectives are Time-To-Failure-of-Strategy (TTFS) as defined by Allegra et al. [47], OS as deaths of all causes, response rate (RR) (investigator evaluated) according to RECIST criteria 1.1 in patients with measurable disease, toxicity, QoL as described by EORTC QLQ-C30, correlation between biomarkers and outcome as well as evaluation of pre-treatment characteristics and geriatric screening tools as predictive markers for efficacy and toxicity.

PFS, TTFS, and OS will be calculated from the date of randomization to the first date of radiological or clinically documented progression to first-line therapy (PD1), first date of documented progression to second line therapy (PD2), or death.

### Statistics

The definition of the target sample size is made based on prior data indicating that median PFS on single agent is 4 months in this patient group, but may be as long as 8 months in patients receiving single agent with bevacizumab [7, 32]. Half of the patients may be candidates for additional bevacizumab, and thus, median PFS for the group of patients receiving single agent therapy may be 6 months. If median PFS for the experimental group (combination therapy, arm B) is 9 months, we need to study 71 experimental patients and 71 control patients to be able to reject the null hypothesis that the experimental and control survival curves are equal with probability (power) 0.8 and a type I error probability of 0.2. To ensure 142 evaluable patients we will include 75 patients in each arm, a total of 150 patients.

PFS and OS data will be estimated by Kaplan-Meier methods and compared with log-rank test. Adverse events will be evaluated in an intention-to-treat-population (all patients who have received at least one course of chemotherapy). The calculations described above are based on the primary end-point. Calculations on the secondary end-points are only hypothesis generating and thus explorative. Our study includes several lines of therapy, which will impair the possibility of finding a significant difference in OS.

## Discussion

This study is conducted to evaluate two treatment strategies in older mCRC patients - full-dose single agent therapy or dose-reduced combination therapy. Patients are not candidates for full dose double or triple combination therapy either because of frailty, very old age, patient preference or because the treating physician recommends less intensive therapy [1], and thus constitute a heterogeneous group. With this study population we have tried to imitate the older population who often challenges physicians in clinical everyday life and where the decision on which treatment regimen to choose is not clear-cut.

As S-1 seems to have a better toxicity profile compared to capecitabine, especially because of a lower incidence of HFS [26], it may be a suitable drug for the study population.

A randomized phase II study, AVEX [32], showed that bevacizumab improved efficacy (RR and PFS) of single agent capecitabine in fit older patients. Therefore it is optional to add bevacizumab to chemotherapy at the discretion of the treating physician in the trial.

Standard treatment in patients with mCRC includes chemotherapy in several lines with an expanding range of treatment options [1]. Therefore we have planned a continuum of care/strategy for patients including both first and second line therapy as part of the trial.

If the study reaches the desired end-point for efficacy, it suggests that reduced combination therapy becomes the standard treatment for patients with mCRC who are not candidates for standard full-dose combination therapy. Future studies will be needed to confirm this and perhaps evaluate the potential benefit of adding bevacizumab to all patients in this particular study population.

SIOG recommends that future research focuses on the ability of screening tools to build clinical pathways and to predict different outcome parameters [48]. We have in the trial chosen to include several geriatric screening tools, which have sensitivity and specificity comparable to a full CGA, but are less time-consuming [48, 49]. Most of the studies performed on geriatric screening tools have been retrospective and performed in patient populations with heterogeneous cancers [39]. We will evaluate the chosen geriatric screening tools in a prospective study design in a population of older mCRC patients in order to predict efficacy and toxicity of the oncological treatment and search for at-risk individuals. If shown to be valid and relevant, the screening tools may in the future be part of routine screening in the treatment decision of geriatric patients [39].

### Trial Status

A total of 120 patients have been enrolled by the 1st of January 2017.

## Abbreviations

5-FU: 5-fluorouracil
CGA: Comprehensive geriatric assessment
CNS: Central nervous system
CRC: Colorectal cancer
G-8: Geriatric-8
GCP: Good Clinical Practice
GFR: Glomerular filtration rate
GS: Handgrip strength
GST: Geriatric screening tools
HFS: Hand-foot-syndrome
HR: Hazard ratio
IRIS: Irinotecan and S-1
mCRC: Metastatic colorectal cancer
NCI-CTC: National Cancer Institute - Common Toxicity Criteria
OS: Overall survival
PD: Progressive disease
PD1: Progressive disease to first-line treatment
PD2: Progressive disease to second line treatment
PFS: Progression-free survival
QoL: Quality of Life
RECIST: Response Evaluation Criteria In Solid Tumors
RR: Response Rate
SIOG: The International Society of Geriatric Oncology
SOx: Oxaliplatin and S-1
TTFS: Time-To-Failure-of-Strategy
TUG: Timed-up-and-Go
VES-13: The Vulnerable Elders Survey
WHO: World Health Organization
